# Oblongifolin M, an active compound isolated from a Chinese medical herb *Garcinia oblongifolia*, potently inhibits enterovirus 71 reproduction through downregulation of ERp57

**DOI:** 10.18632/oncotarget.7122

**Published:** 2016-02-01

**Authors:** Mengjie Wang, Qi Dong, Hua Wang, Yaqing He, Ying Chen, Hong Zhang, Rong Wu, Xinchun Chen, Boping Zhou, Jason He, Hsiang-Fu Kung, Canhua Huang, Yuquan Wei, Jian-dong Huang, Hongxi Xu, Ming-Liang He

**Affiliations:** ^1^ School of Pharmacy, Shanghai University of Traditional Chinese Medicine, Shanghai, China; ^2^ Shenzhen Research Institute, The Chinese University of Hong Kong, Shenzhen, China; ^3^ Stanley Ho Center for Emerging Infectious Diseases, The Chinese University of Hong Kong, Hong Kong, China; ^4^ Shenzhen Center for Disease Control and Prevention (Shenzhen CDC), Shenzhen, China; ^5^ Institute of Infectious Diseases, The 3rd Peoples’ Hospital of Shenzhen, Shenzhen, China; ^6^ State Key Laboratory of Biotherapy and Cancer Center, West China Hospital, Sichuan University, Chengdu, China; ^7^ School of Biomedical Sciences, The University of Hong Kong, Hong Kong, China; ^8^ College of Letter and Sciences, University of California at Berkeley, Berkeley, CA, USA; ^9^ Department of Biomedical Sciences, City University of Hong Kong, Hong Kong, China

**Keywords:** enterovirus 71, Oblongifolin M, ERp57, antiviral

## Abstract

There is no effective drug to treat EV71 infection yet. Traditional Chinese herbs are great resources for novel antiviral compounds. Here we showed that *Oblongifolin M* (OM), an active compound isolated from *Garcinia oblongifolia*, potently inhibited EV71 infection in a dose dependent manner. To identify its potential effectors in the host cells, we successfully identified 18 proteins from 52 differentially expressed spots by comparative proteomics studies. Further studies showed that knockdown of ERp57 inhibited viral replication through downregulating viral IRES (internal ribosome entry site) activities, whereas ectopic expression of ERp57 increased IRES activity and partly rescued the inhibitory effects of OM on viral replication. We demonstrated that OM is an effective antiviral agent; and that ERp57 is one of its cellular effectors against EV71 infection.

## INTRODUCTION

Enterovirus 71 (EV71), a member of the *Picornaviridae* family, is a non-enveloped single-stranded RNA virus. It is the major causative agent of repeated outbreaks of hand, foot and mouth disease (HFMD) [1, 2]. In severe cases, especially those among in infants and children, EV71 infection causes severe neurological complications such as aseptic meningitis, brain stem encephalitis, pulmonary edema, poliomyelitis-like paralysis and eventual death. In the last decade, the continuous outbreaks of EV71 in Asia-Pacific region have caused considerable deaths [3]. More than 7 million HFMD cases were reported in China between 2008 and 2012, of which 2457 were fatal [4, 5]. No effective antiviral drug is currently used for treating EV71 infection [6]; therefore, there is an urgent need for effective agent against EV71 infection.

Chinese Medical herbs are a great reservoir of active compounds against microbial infections [7–14]. Plants generate a great deal of compounds to eliminate or limit microbe invasions. The family *Guttiferae* contains more than 450 species distributed over regions mostly in Asia, southern Africa and Western Polynesia. Many bioactive compounds, such as prenylated xanthenes, benzophenones, biflavonoids, and polycyclic polyprenylated acylphloroglucinols, have been isolated from different family members [15–23]. They exhibit various biological activities including antibacterial, antifungal, antioxidant, anti-inflammatory and anticancer effects [15–23], despite the underlining mechanisms being poorly understood.

With the growing number of natural antiviral compounds identified, a promising option would be to find new compounds from medical herbs to combat EV71 infection [24–26]. Instructed by bioactivity-guided isolation, a new isoprenyl benzophenone derivative *Oblongifolin M* (compound M from *Oblongifolia*, OM), has been isolated from medical herb *Garcinia oblongifolia* [27]. In this study, we investigated its antiviral activity against EV71 infection and the underlying mechanisms through comparable proteomics studies.

## RESULTS

### Protection of cytopathic effects (CPE) caused by EV71 infection

**Figure 1:**
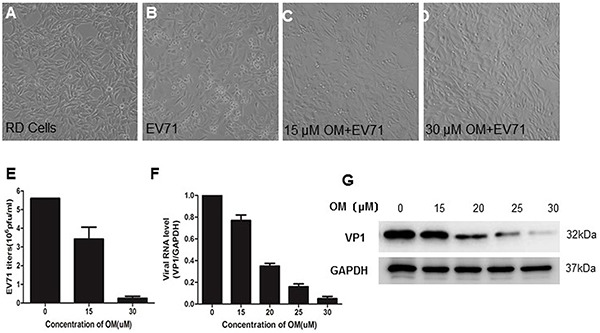
The inhibitory effects of OM against EV71 infection OM protected cytotoxic effects (CPE) from EV71 infection in RD cells **A.** to **D.** RD cells were pretreated without (A, B) or with OM (C, D) for 4 hours before EV71 infection at a MOI of 1. Photos were taken 12 hours post-infection (p.i.). **E.** OM inhibited EV71 reproduction. The virions in the culture supernatant were harvested 12 hours p.i. and the viral titer was determined by TCID_50_ assays. **F.** OM inhibited EV71 replication. 12 hous p.i., the intracellular RNA was isolated, and viral genomic RNA (primers targeting VP1 gene) and cellular mRNA of GAPDH genes were quantitated by qRT-PCR. The viral genomic RNA level was normalized with the copy number of mRNA of GAPHD. The mean value of VP1/GAPDH ratio was set as 1 in control. **G.** The viral VP1 protein was decreased by OM in a dose dependent manner. The cells were harvested 12 hours p.i. at MOI of 1, and cell lysate was applied for Western blot assays.

The antiviral effect of OM was tested in Rhabdomyosarcoma (RD) cells by CPE assays. As shown in Figure 1A, the RD cells without viral infection were flat with spindle-like sharps and attached well on the surface of culture dishes. When cells were infected with EV71 at a multiple of infection (MOI) of 1, many cells showed CPE. They became round, detached from the surface of culture dishes and floated away 12 hours post-infection (p.i.) (Figure 1B). When the cells were pretreated with 15 μM of OM 4 hours prior to EV71 infection, most cells were healthy with only a few exhibiting CPE (Figure 1C), an indication that the infected cells were significantly protected from CPE. Surprisingly, almost all EV71-infected cells were healthy when pretreated with 30 μM of OM (Figure 1D). Similar results were also obtained from HEK 293 cells and Hela cells (data not shown). The reduction of EV71-induced CPE by 50% (IC_50_) was determined byusing GraphPad Prism5. As shown in Table 1, the IC_50_ of OM was 2.38±0.79 μM. Cell viability was employed to determine the toxicity of OM in RD cells by MTT (3-(4,5-dimethyl-2-thiazolyl)-2,5-diphenyl-2-H-tetrazolium bromide) assays after treating RD cells with OM for 24h. Compared with untreated cells, cell viability was not obviously affected by OM at a concentration of 50 μM. The CC_50_ (concentration of OM required for 50% cell kill) of the uninfected cells was 83.87±1.12 μM. The selectivity of OM on RD cells was 35.24 (Table 1), indicating OM's potential to be an effective antiviral agent against EV71 infection.

**Table 1:** Cytotoxic, antiviral activity and selectivity index of OM against EV71

Compound	Cytotoxicity	Antiviral activity^a^	Selectivity Index
OM	CC_50_(μM)	IC_50_(μM)	SI (CC_50_/IC_50_)
	83.87±1.12^b^	2.38±0.79^b^	35.24

a RD cells were infected with EV71 at a MOI of 0.01 after treatment with serial dilution of OM for 4 h.

b Values obtained from nonlinear regression analysis using GraphPad Prism5.

Values represent the mean ± SD of three independent experiments.

### Inhibition of EV71 propagation

To further determine OM's antiviral potency, we titrated the viral titers in the culture media. As shown in Figure 1E, the mean value of viral titers was 5.6 × 10^6^ pfu/ml in the control group. After treatment of the cells with 15 μM of OM, the viral titers significantly decreased to 3.42 × 10^6^ ± 6.4 × 10^5^ pfu/ml. When the concentration of OM was increased to 30 μM, surprisingly, the viral titers dramatically dropped by over 90% to 2.5 × 10^5^ ± 1.1 × 10^5^ pfu/ml. The antiviral effects were further validated by measuring the intracellular viral genomic RNA copies and viral protein levels. Twelve hours after infection, the copy number of viral genomic RNA decreased by 23%, 65%, 84% and 95% after cells were treated with OM at concentrations of 15, 20, 25, and 30 μM, respectively (Figure 1F). Consistently, the levels of intracellular VP1 protein were significantly inhibited by OM in a dose dependent manner (Figure 1G).

### The effects of OM on protein profiles

**Figure 2:**
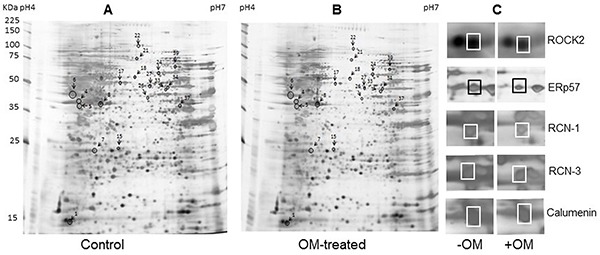
Protein profile differences between Control and OM-treated RD cells **A.** and **B.** representative silver-stained 2-DE maps of proteins from DMSO and 30 μM of OM (resolved in DMSO) treated RD cells. Fifty two differential expressed spots were picked up for MALDI-TOF-MS and MS/MS analysis. The spots of eighteen differently expressed proteins identified were marked. **C.** The enlarged sections of the 2-DE map. Spot 4, RCN-3; spot 5, RCN-1; spot 6, calumenin; and spot 17, ERp57.

To investigate the potential antiviral mechanisms of OM against EV71 infection, we employed comparable proteomics studies. Proteins from RD cells treated with DMSO (control) or 30 μM of OM were extracted and resolved by 2-DE analysis 48 hours post-treatment. Figure 2 shows a representative pair of silver-stained 2-DE maps between two samples from three pairs of gels (Figure 2A and 2B). After comparison, 52 spots showed significant (with over 2-fold, p < 0.05) difference. The individual protein spot was extracted and digested for MALD-TOF MS and MS/MS analysis. Typical images of five paired spots were cropped and enlarged as shown in Figure 2C. We successfully identified 18 proteins with significant differential expression. Among them, eight proteins were markedly up-regulated whereas ten were down-regulated in the OM-treated RD cells as compared to the control group. The characteristics of proteins (including name, abbreviation, NCBI accession number, theoretical molecular mass, pI, peptide count, protein score (confidence interval in percent), number of unmatched masses, sequence coverage, fold change, and functions) are listed in Table 2. Functionally, they can be categorized into six categories: structural molecular activity (17%), metabolism (39%), cytoskeleton and transport proteins (11%), gene transcription associated proteins (6%), heat shock proteins and chaperones (17%), and cell proliferation, metastasis, and signal transduction proteins (11%).

**Table 2:** Differentially expressed proteins identified by 2-DE/MS analysis in RD cells treated without/with OM (At a 2-fold difference cutoff in intensity, xx spots were selected for further MALD-TOF-MS/MS analysis, 18 proteins were finally identified)

Spots No.^a^	Protein name	Abbreviation	NCBI accession no.	Theoretical molecular mass(Da)	PI	Protein core (CI)	Masses matched/ searched	Sequencecoverage (%)	‘-Fold change^b^
**Structural molecule activity**									
1	Rho-associated protein kinase 2	ROCK2	O75116	161939	5.75	69	9/13	5	11.7↓
24	Prelamin-A/C	LMNA	P02545	74380	6.57	97	15/36	22	4.1↓
26	Annexin A7	ANXA7	P20073	52991	5.52	71	8/21	15	3.7↑
**Metabolism**									
4	Reticulocalbin-3	RCN3	Q96D15	37470	4.74	94	8/27	32	18.2↓
5	Reticulocalbin-1	RCN1	Q15293	38866	4.86	115	12/34	34	15.3↓
6	Calumenin	CALU	O43852	37198	4.47	80	9/30	26	4.3↓
7	Proteasome subunit alpha type-3	PSMA3	P25788	28643	5.19	60	6/26	20	3.8↑
15	N(G),N(G)-dimethylarginine dimethylaminohydrolase 2	DDAH2	O95865	29911	5.66	59	6/28	18	3.6↑
21	Mitochondrial inner membrane protein	IMMT	Q16891	84025	6.08	117	14/28	16	3.3↓
37	26S protease regulatory subunit 10B	PSMC6	P62333	44430	7.1	58	6/14	13	3.3↑
**Cytoskeleton and transport proteins**									
8	Actin, cytoplasmic 1	ACTB	P60709	42052	5.29	111	9/29	26	3.8↑
22	Vinculin	VCL	P18206	124292	5.5	96	14/27	11	3.0↑
**Gene transcription**									
39	Far upstream element-binding protein 1	FUBP1	Q96AE4	67690	7.18	110	11/21	17	3.1↑
**Heat shock proteins and chaperones**									
17	Protein disulfide-isomerase A3	ERp57	P30101	57146	5.98	76	11/31	17	13↓
18	T-complex protein 1 subunit alpha	TCP1	P17987	60819	5.8	77	9/24	16	3.4↓
27	T-complex protein 1 subunit beta	CCT2	P78371	57794	6.01	121	13/29	22	3.9↓
**Cell proliferation, metastasis, and signal transduction**									
33	Septin-11	SEPT11	Q9NVA2	49652	6.36	114	12/22	22	8.5↑
34	Fascin	FSCN1	Q16658	55123	6.84	103	10/24	18	10.8↓

a Spot numbers are shown in Figure 2.

b The spot intensities were quantified using PDQuest software (Bio-Rad). The average -fold change of spot intensity for each protein was calculated from three independent experiments (DMSO-treated RD versus OM-treated RD). ↑, increase; ↓, decrease.

### Validation of differentially expressed protein by western blot assay

**Figure 3:**
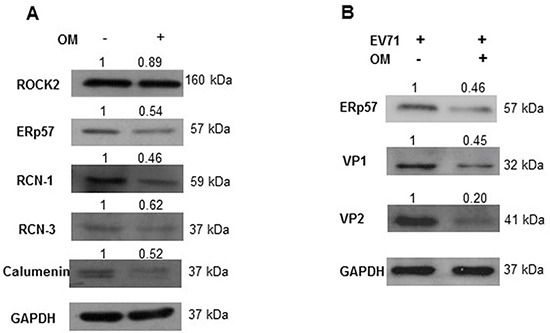
The suppression of ERp57 expression by OM **A.** RD cells were treated with OM at 30 μM for 48 hours, and cell lysates were harvested and applied for Western blot assays. As shown, ERp57, RCN-1, RCN-2 and Calumenin were markedly down-regulated by OM treatment. **B.** Cells were pretreated with 30 μM of OM for 4 hours, and then infected with EV71 at MOI of 1 for 12 hours. As shown, OM suppressed the expression of ERp57, and viral proteins VP1 and VP2. The relative density value of each band to GAPHD was set as 1 in the control group.

We carried out Western blot assays to confirm the differentially expressed protein levels after we treated the RD cells with OM for 48 hours. Among five selected candidate proteins, four displayed similar expression patterns as shown in the 2-DE assays. The protein levels of ERp57, RCN-1, RCN-3, and CALU significantly decreased after OM treatment (Figure 3A).

ERp57 drew our particular attention because it is crucial for the entry of SV40 and murine rotavirus [28, 29]. To further observe the correlation between the expression levels of ERp57 and EV71 viral proteins, RD cells were treated with or without 30 μM of OM for 4 hours, and then infected with EV71 (MOI 1). As shown in Figure 3B, OM not only suppressed ERp57 expression, but also significantly decreased viral VP1 and VP2 levels. These results indicated that ERp57 might involve the life cycle of EV71.

### Suppression of EV71 by knockdown of ERp57

**Figure 4:**
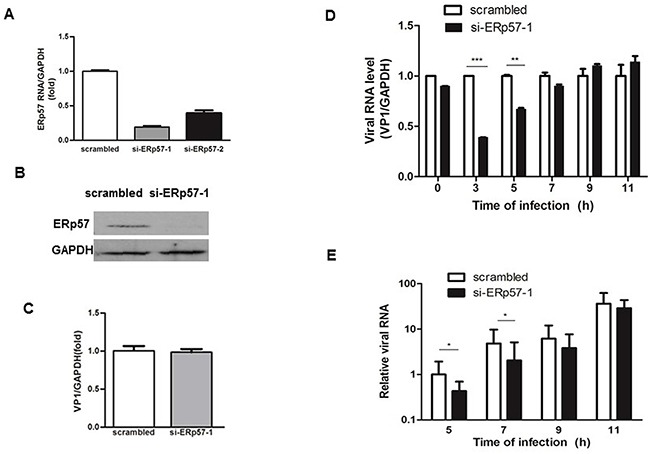
Inhibition of EV71 infection by Knockdown of ERp57 at early stage **A.** Specific siRNAs targeting ERp57 were transfected into RD cells, and the effect of knockdown was determined by qRT-PCR 48 hours post-transfection. **B.** The ERp57 protein level detected by si-ERp57-1 knockdown. **C.** Knockdown of ERp57 did not affect EV71 entry. Forty eight hours post-transfection with siRNAs, cells were infected with EV71 at MOI of 1 for 1 hour (time point was set as 0 hour), and cells were washed with PBS for three times. The intracellular viral RNA level was determined by qRT-PCR. The mean value of VP1/GAPDH ratio was set as 1 in control. **D.** and **E.** The intracellular (D) and extracellular (E) viral RNA levels upon ERp57 knockdown at different time points. The total RNA was isolated from infected cells and corresponding media at different time points, and quantified by quantitative RT-PCR. At time point 0 hour, the intracellular viral RNA level (virions just entered the cells) were quantitated by qRT-PCR *, *p* < 0.05; **, *p* < 0.01; ***, *p* < 0.001.

We tested the effects of ERp57 knockdown on EV71 infections. As shown in Figure 4A, both si-ERp57-1 and ERp57-2 effectively reduced the mRNA level of ERp57. As si-ERp57-1 was more effective on knocking down ERp57, si-ERp57-1 was chosen for further experiments in our study. Consistent with the reduction of mRNA level, ERp57 protein level was also significantly decreased by si-ERp57-1 (Figure 4B). We further examined if knockdown of ERp57 would affect virus entry. RD cells were first transfected with either scrambled or si-ERp57-1 for 48 hours, and then infected with EV71 at MOI of 20 for 1 hour on ice. The infected cells were washed with PBS for three times to remove potential free viruses that had not entered the cells. We employed qRT-PCR to measure the intracellular viral RNA levels. Our data showed that the entry of EV71 had not been affected by ERp57 knockdown (Figure 4C). To further investigate if ERp57 knockdown would affect EV71 replication or reproduction, we first measured the intracellular viral RNA levels at different time points. We showed that ERp57 knockdown significantly decreased the intracellular viral RNA levels at 3 and 5 hours p.i. (Figure 4D), and markedly reduced the secreted virions in the culture media collected at 5 and 7 hours p.i. (Figure 4E). These results indicate that ERp57 was involved in the early phase of viral life cycle.

### Inhibition of IRES activity by OM and ERp57

**Figure 5:**
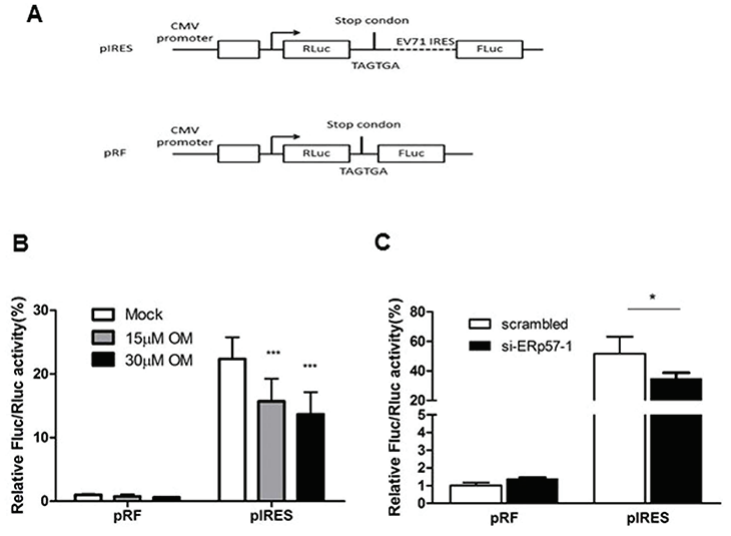
Inhibition of EV71 IRES activity by OM or knockdown of ERp57 **A.** Schematic diagram of plasmids pIRES and pRF for measuring IRES activity. **B.** OM inhibited IRES activity in a dose dependent manner. Cells were transfected with pIRES or pRF for 24 hours, and then treated without or with OM for 12 hour followed by luciferase assays. **C.** Knockdown of ERp57 reduced IRES activity. Cells were transfected with scramble siRNA or si-ERp57-1. Twenty four hours post-transfection, the cells were then transfected with pIRES or pRF for another 24 hours and applied for luciferase assays. The ratio of FLuc to RLuc activity was set as 1 when cells were transfected with pRL reporter plasmid in the control group (without OM treatment or transfected with scrambled siRNA). *, *p* < 0.05; ***, *p* < 0.001.

Our previous study showed that active replication of EV71 occurs just after uncoating (3 to 6 hours p.i.) [30]. After uncoating, the first and key event is viral protein translation driven by IRES [31]. Therefore, we hypothesized that OM may inhibit EV71 infection by down-regulation of IRES activity through ERp57. To test this hypothesis, we generated two constructs to express reporters *Renilla* luciferase (RLuc) and *Firefly* luciferase (FLuc). As shown in Figure 5A, two continuous stop codes (TGA TGA) were inserted between RLuc and FLuc genes in pRF plasmid; while the IRES sequence of EV71 was inserted between RLuc and FLuc genes in pIRES plasmids. In both constructs, the CMV promoter drives RLuc expression. FLuc translation is stopped when mRNA is transcribed from pRF, whereas the translation would be successful from that of pIRES. Hence, the IRES activity can be measured and expressed by the ratio of FLuc to RLuc activities.

We showed that OM did not affect the ratio of FLuc/RLuc when cells were transfected with pRF plasmid; however, the ratio of FLuc/RLuc decreased after cells were transfected with pIRES and treated with OM. The inhibition of IRES activity was in a dose-dependent manner (Figure 5B). As expected, when ERp57 was knocked down, the IRES activity of EV71 markedly decreased (Figure 5C), correlating with the inhibitory effects of OM.

### Restoration of OM-suppressed IRES activity by ectopic expression of ERp57

**Figure 6:**
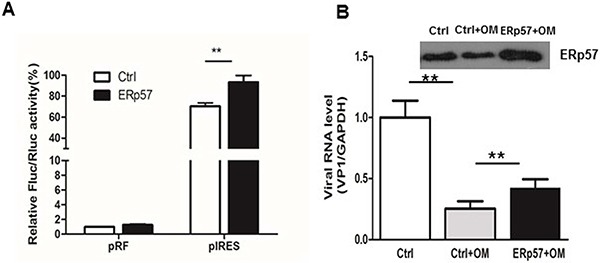
Ectopic expression of ERp57 enhanced IRES activity **A.** Ectopic expression ERp57 increased the reporter expression driven by IRES; **B.** Ectopic expression of ERp57 partly rescued OM's inhibitory effect on IRES. **, *p* < 0.01.

We constructed an expression plasmid to express human ERp57 in HEK 293 cells. As shown in Figure 6A, the ectopic expression of ERp57 significantly stimulated the IRES activity of EV71. Furthermore, we examined if ectopic expression of ERp57 would restore the viral replication that had been inhibited by OM. ERp57 was ectopically expressed in RD cells and pretreated with OM at 30 μM for 4 hours. The cells were then infected with EV71 at MOI 1. We showed that the viral RNA level decreased to 25% that of the control 5 hours p.i., whereas the viral RNA level was restored to that 53% of the control upon overexpression of ERp57. Our results demonstrated that ERp57 partly restored the IRES activity suppressed by OM.

## DISCUSSION

There is no effective drug to treat EV71 infection [31–33]. In this paper, we showed that an effective compound OM isolated from *Garcinia oblongifolia* displayed potent anti-EV71 activitythrough downregulation of ERp57, an important host factor in the early phase of EV71 life cycle.

Host proteins are ideal targets for antiviral drug development because targeting host proteins would avoid or delay the occurrence of drug resistance due to fast mutagenesis of viruses. ERp57, a chaperon protein involved in catalyzing bisulfate bond exchange for correct protein folding in ER stress, has been shown a crucial role in the entry of DNA virus SV40 [28]. Besides virus entry, it is possible that ERp57 may exhibit other functions in the life cycle of other viruses. As an RNA virus, EV71 has a life cycle that is completely different from that of SV40. An important question is whether ERp57 exhibits a similar function in the life cycle of EV71. To address this question, we first investigated if the viral entry of EV71 would be affected by knockdown of ERp57. To our surprise, the depletion of ERp57 did not affect virus entry into the host cells (Figure 4C). Interestingly, we found that the virus replication was significantly suppressed at 3 to 5 hours p.i. after ERp57 knockdown in the host RD cells (Figure 4D). Accordingly, the secreted virions evidently decreased at 5 to 7 hours p.i. (Figure 4E). In our previous study, we have shown that EV71 undergoes the fast replication phase from 3 to 6 hours p.i. in RD cells, and carries out fast package from 6 to 9 hours p.i. (package phase) and secretion initiation [30]. Our results suggested that ERp57 involves mainly in the early phase of viral life cycle.

The first event of viral activity is IRES-mediated translation in generating proteins required for viral RNA replication after uncoating of virus in the host cells. We hypothesized that ERp57 may affect viral translation through regulating viral IRES activities. Our results showed that knockdown of ERp57 significantly reduced the viral IRES activities (Figure 5B). Intriguingly, OM also suppressed the IRES activity in a dose dependent manner (Figure 5C), demonstrating a correlation between drug effects and ERp57 functions. To further validate our findings, we conducted gain-of-function studies. ERp57 was ectopically expressed in host cells to test its effects on IRES activity. As expected, results from this study exhibited an increase of IRES activity by ectopic ERp57 expression (Figure 6A). This suggests that OM may affect ERp57 functions in an indirect or direct manner. We have noted that ectopic expression of ERp57 partly rescued the inhibition of early replication of EV71 (Figure 6B), indicating that in addition to ERp57, there must be other host factors contributing to the inhibitory effects of OM on EV71 reproduction. As shown in Table II, 10 proteins were down-regulated and 8 proteins were upregulated by OM. Whether these proteins contribute to the viral inhibition of OM should be investigated in future studies. Although several other compounds isolated from *Garcinia oblongifolia* (e.g., Oblongifolia L or P) share similar structure to OM, it antiviral effects against EV71 was much weaker (data not shown). Further investigating the underling mechanism may help us to develop OM derivatives with more potent anti-EV71 activity.

In summary, we showed that OM, an active compound isolated from a traditional Chinese herb, potently inhibited EV71 infection. By employing comparative proteomics studies, we identified that ERp57 was an effector of OM. Knockdown of ERp57 inhibited viral replication through downregulating viral IRES activities, whereas ectopic expression of ERp57 increased IRES activity and partly rescued the inhibition of viral replication by OM. Our results demonstrated that OM inhibited the early replication of EV71 partly through downregulating ERp57. OM could potentially be further developed into a therapeutic drug for treating EV71 infections, and ERp57 may serve as a target for developing host-based antiviral drugs against EV71 infection.

## MATERIALS AND METHODS

### Chemicals and antibodies

Chemicals were purchased from Sigma (St. Louis, MO, USA). Oblongifolin M (OM) was previously identified and isolated with purity over 98% [27]. Commercially available antibodies were purchased from different companies. Antibody against VP1 was from Abnova (Cat. No. PAB7630-B01P), VP2 specific antibody was from Millpore (Cat. No. MAB979), rabbit polyclonal anti-RCN1 (Cat No. ab129796), anti-RCN3 (Cat No. 134228), anti-CALU (Cat. No. ab118308), and anti-ROCK2 (Cat. No. ab66320) were from Abcam, anti-ERp57 (Cat. No. H-220) and anti- GAPDH (Cat. No. sc-32233) specific antibody and all the corresponding secondary antibodies were purchased from Santa Cruz Biotechnology.

### Cell culture, virus propagation and compound

Rhabdomyosarcoma cells (RD, ATCC accession no. CCL-136) were maintained in Dulbecco's modified Eagle's medium (DMEM) containing 10% (v/v) fetal bovine serum (FBS, HyClone) with 100 U/ml penicillin and 100 μg/ml streptomycin, at a humidified condition of 5% CO_2_ at 37°C. EV71 (SHZH98 strain; GenBank accession number AF302996.1) [30, 44, 45] was propagated on 90% confluent monolayer cells in DMEM with 2% FBS. When about 80% of the cells exhibited CPE, culture fluid was collected, centrifuged, filtrated and stored in −80°C until use. The viral titer was determined by the end-point dilution assay of median tissue culture infective dose (TCID_50_).

### CPE assay

Antiviral activity of OM was evaluated by CPE assays. Approximately 20,000 RD cells per well were laid in 96-well plate for 24 hours at 37°C to reach monolayer, then treated with OM at different concentrations and infected by EV71 at MOI of 0.01 or 1 (Supplementary Table S1). CPE was monitored from time to time under a phase-contrast microscope and recorded by a CCD camera. The concentration required for the tested compound to reduce the EV71-induced CPE by 50% (IC_50_) was determined by using the Forecast function of Microsoft Excel for all experiments. Data were shown as mean values with standard deviations from three independent assays. Selectivity index (SI) is calculated by the ratio of CC_50_ to IC_50_.

### Cytotoxicity test

Cytoxicity of OM was evaluated by cell viability assays. RD cells (about 20,000 cells) were set in each well of a 96-well plate overnight to reach monolayer and exposed to serial dilution of OM for 24 hours. Cell viability was measured by the MTT method as described previously [46, 47]. The CC_50_ was calculated.

### Western blot

Cells were lysed in radioimmunoprecipitation assay (RIPA) buffer (50 mM Tris-HCl, pH 7.5, 150 mM NaCl, 1mM EDTA, 1% Triton X-100, 0.1% SDS, 1×Roche protease inhibitor cocktail) with occasional vortex. The cell lysates were then centrifuged to remove debris at 14,000 rpm for 20 min at 4°C. The concentration of proteins in the lysates was determined by Bradford assay (Bio-Rad). Equal amounts of total protein for each sample was loaded and separated by 8% to 12% SDS-PAGE and then transferred onto polyvinylidene difluoride (PVDF) membranes (Amersham Biosciences). Membranes were blocked with 5% skim milk in TBST (20 mM Tris-HCl, pH 7.4, 150 mM NaCl, 0.1% Tween 20) for 1 h and incubated with specific antibodies. GAPDH was served as the loading control. Target proteins were detected with corresponding secondary antibodies (Santa Cruz Biotechnology), visualized with a chemiluminescence detection system (Amersham Biosciences). Each immunoblot assay was carried out at least three times as previously reported [44].

### Real-time RT-PCR

The total cellular RNA was extracted using TRIzol reagent (Invitrogen) according to the manufacturer's instructions. The virion RNA in the culture media was isolated using a viral RNA isolation kit (Qiagen). RNA was than reverse transcribed into cDNA using a reverse transcription system (Promega). Quantitative reverse transcription-PCR (qRT-PCR) was carried out by using an ABI 7500 Real-Time PCR system with SYBR Premix Ex Taq (Takara). The PCR was set up under the following thermal cycling conditions: 95°C for 30 s, followed by 40 cycles of 95°C for 5 s and 60°C for 34 s. The threshold cycle (CT) value was normalized to that of glyceraldehyde-3-phosphate dehydrogenase (GAPDH) [47, 48]. The qRT-PCR was performed by using the following primer pairs: GAPDH, 5′-GATTCCACCCATGGCAAATTCCA-3′ (forward) and 5′-TGGTGATGGGATTTCCATTGATGA-3′ (reverse); EV71, 5′-GCAGCCCAAAAGAACTTCAC-3′and 5′-ATTTCAGCAGCTTGGAGTGC-3′; for ERP57, 5′-GTGCTAGAACTCACGGACGA-3′ and 5′-GCTGCAGCTTCATACTCAGG-3′. All samples were run in triplicates, and the experiment was repeated three times. The relative mRNA level of each target gene was expressed as fold change relative to the value of the corresponding control.

### Two-dimensional gel electrophoresis and image analysis

RD cells were treated with 30 μM of OM (in DMSO) or an equal amount of DMSO for 48 hours. The cells were washed with wash buffer (10 mM Tris, 250 mM sucrose, pH 7.0) six times to remove salt ions thoroughly, lysed with lysis buffer (8 M urea, 2 M thiourea, 2% CHAPS, 1%Nonidet P-40, 2 mM tributylphosphine, 1× Roche Applied Science protease inhibitor mixture, 1× nuclease mixture, 1 mM PMSF, 2% IPG buffer), and then left on ice for 45 min with occasional vortex. The lysates were centrifuged (14,000 g for 15 min) at 4°C, the supernatants were collected and stored at −80°C until use. Protein concentrations were measured by Bradford assay (Bio-Rad). Isoelectric focusing was conducted with 13-cm precast IPG strips (pH 4–7, linear; GE Healthcare) by an Ettan IPGphor II IEF System (Amersham Biosciences). The IPG strips containing 150 μg of protein samples were rehydrated for 10 h at 30 V with 250 μl rehydration buffer (8 M urea, 2% CHAPS, 0.4% DTT, 0.5% IPG buffer, 0.002% bromphenol blue). We then focused the rehydrated strips using a stepwise voltage increment program: 500 and 1000 V for 1 h each and 8000 V afterward until 64 kV-h. After IEF, we incubated the isoelectrically focused strips in an equilibration buffer (6 M urea, 1% DTT, 2% SDS, 30% glycerol, 0.002% bromphenol blue, 50 mM Tris-HCl, pH 6.8) for 15 min with gentle agitation, then incubated the strips for another 15 min in the same buffer containing 2.5% iodoacetamide without DTT. Then we loaded the equilibrated strips onto 12.5% SDS-polyacrylamide gels, run at 15 mA/gel for 30 min and then 30 mA/gel until the dye fronts reached the bottoms of the gels. We stained the gels by modified silver staining, which was compatible with MS analysis. Gels were scanned with a calibrated GS-800 scanner (Bio-Rad), and the intensity of spots was calculated and compared by using Quantity One and PDQuest 2-D analysis software (version 8.0; Bio-Rad) as described previously [35]. A 2-fold increase/decrease (DMSO-treated versus OM-treated RD cells) of spot intensities was set as the threshold for indicating significant changes.

### MALDI-TOF/TOF MS and MS/MS spectrometry analysis

Selected spots were manually cut from the gels, destained, washed and dried completely by vacuum and digested with 10 μg/ml trypsin (Promega) in 25 mM ammonium bicarbonate, pH 8.0 for 16–18 h at 37°C. The supernatants containing tryptic peptides were collected. We mixed 2 μl of peptide solution with 0.6 μl of matrix (6 mg/ml α-cyano-4-hydroxylcinnamic acid in 45% ACN and 0.2% TFA) before spotting onto the MALDI plate. The samples were applied for mass spectrometric analyses by using a MALDI-TOF/TOF tandem mass spectrometer Ultraflex III proteomics analyzer (Bruker Daltonics). We obtained mass spectra from 2000 laser shots with an accelerating voltage of 20 kV with mass ranges of 700–4000 m/z in a positive ion reflectron mode and mass errors of less than 100 ppm by using the FlexControl (version 3.3.108.0, Bruker Daltonics). We obtained MS/MS spectra by collecting 3000 laser shots with a default calibration. The detected MS/MS peaks were set on a minimum S/N ratio ≥3 and cluster area S/N threshold ≥15 with smoothing, as described previously [49]. The information from combined MS and MS/MS analysis was applied for protein identification search against SwissPort database (545388 protein sequences; released on June 23, 2014) by the Mascot search engine (version 2.2.04; Matrix Science) and BioTools software (version 3.5; Bruker Daltonics). The search parameters were defined as follows: taxonomy of Homo sapiens, trypsin digest with a maximum of one missed cleavage, fixed modification of cysteine carbamidomethylation, variable modification of methionine oxidation, monoisotopic peptide mass (MH^+^), mass range unrestricted, pI of 0–14, precursor tolerance of 100 ppm, and MS/MS fragment tolerance of 0.5 Da. Before database searching, we removed the known contaminant ions from the spectra, which correspond to human keratin and trypsin autolysis peptides. The top 20 hits for each protein search were reported. The protein candidate was reported only when it met the maximum number of matched peptides and a pI value nearest to the observed value. Each isoform of a protein family, which was identified, was considered to be a distinct protein for analysis, as described previously [49].

### siRNA synthesis and transfection

Specific siRNA for ERp57 (siERp57-1) was purchased from QIAGEN (Cat. No. SI02654771). An in-house designed siRNA (si-ERp57-2) and nonspecific siRNA (Scrambled siRNA) were synthesized by GenePharma Co. (Shanghai). Scrambled siRNA (5′ UUC UCC GAA CGU GUC ACG UTT ACG U 3′), which displays no homology to EV71 or the human genome, was used as the negative control (NC) in this study. RD cells were transfected with siRNAs with HiPerFect transfection reagent (Qiagen) according to the manufacturer's instructions. The efficiency of knockdown was measured by qRT-PCR and Western blot assays.

### EV71 infection, quantification of intracellular and extracellular viral genomic RNA

RD cells were preincubated with OM for 4 hours or transfected with siRNAs for 48 hours, the cells were washed twice with phosphate buffered saline (PBS) and infected with EV71 at MOI of 1. Time was set to zero after adsorption for 1 h. The culture media were removed and cells were washed twice with PBS to remove unattached virus before adding 0.2 mL of DMEM medium containing 2% FBS to each well. To quantify the intracellular viral RNA or extracellular viral RNA in the virions, total RNA was isolated from infected cells or culture media at different time-points and further subjected to qRT-PCR assays [30].

### Luciferase activity assay

Cells (HEK 293) were set in 12-well dishes overnight, then transfected with 100 ng of pRF or pIRES plasmid. 24 hours post-transfection, cells were treated without or with OM at a final concentration of 15 or 30 μM for 12 hours. The cells were then harvested and cell lysates were applied for luciferase assays using a Dual Luciferase Assay System (Promega, WI) in accordance with manufacturer's instructions.

### Statistical analysis

Results were expressed as mean ± standard deviation (SD). All statistical analyses were carried out with SPSS, version 14.0 software (SPSS Inc.). Two-tailed Student's t test was applied for two group comparisons. A p value <0.05 was considered statistically significant.

## SUPPLEMENTARY TABLE


