# Supplementary material for: Oblongifolin M, an active compound isolated from a Chinese medical herb Garcinia oblongifolia, potently inhibits enterovirus 71 reproduction through downregulation of ERp57
Source: Oncotarget. 2016 Feb 1;7(8):8797–808. doi: 10.18632/oncotarget.7122 (PMC4891005; doi:10.18632/oncotarget.7122)
Supplement: Supplementary file 1 [file oncotarget-07-8797-s001.pdf]

SUPPLEMENTARY TABLE

Supplementary Table S1: Antiviral activity of OM against EV71 (strain; SHZH98), MOI of 1

Testing compound	CC_{50}[a](μM)	IC_{50}[b](μM)	IC_{90}[c](μM)	SI[c]
OM	86.60±1.68[d]	17.55±1.39[d]	24.72±0.20[d]	4.93

[a]Cytotoxicity (CC50) was determined by MTT assay on RD cells at 24h.
[b]Antiviral activity (IC_{50}) was tested by real-time PCR assay to quantify the intracelluar viral RNA level on RD cells at MOI of 1, p.i. 12h.
[c]Selectivity Index (SI) is the ratio of CC50 to IC50.
[d]Values represent the mean ±SD of three independent experiments.
